# Serine Hydroxymethyltransferase from the Cold Adapted Microorganism *Psychromonas ingrahamii*: A Low Temperature Active Enzyme with Broad Substrate Specificity

**DOI:** 10.3390/ijms13021314

**Published:** 2012-01-25

**Authors:** Sebastiana Angelaccio, Rita Florio, Valerio Consalvi, Guido Festa, Stefano Pascarella

**Affiliations:** Department of Biochemical Sciences “A. Rossi Fanelli”, University of Rome “La Sapienza”, P.le Aldo Moro 5, Roma 00185, Italy; E-Mails: Rita.Florio@uniroma1.it (R.F.); Valerio.Consalvi@uniroma1.it (V.C.); festaguido@gmail.com (G.F.); Stefano.Pascarella@uniroma1.it (S.P.)

**Keywords:** cold adaptation, serine hydroxymethyltransferase, structural flexibility, temperature dependence of enzyme activity, catalytic promiscuity, pyridoxal-5′-phosphate, cofactor-mediated stabilization, psychrophiles, *Psychromonas ingrahamii*

## Abstract

Serine hydroxymethyltransferase from the psychrophilic microorganism *Psychromonas ingrahamii* was expressed in *Escherichia coli* and purified as a His-tag fusion protein. The enzyme was characterized with respect to its spectroscopic, catalytic, and thermodynamic properties. The properties of the psychrophilic enzyme have been contrasted with the characteristics of the homologous counterpart from *E. coli*, which has been structurally and functionally characterized in depth and with which it shares 75% sequence identity. Spectroscopic measures confirmed that the psychrophilic enzyme displays structural properties almost identical to those of the mesophilic counterpart. At variance, the *P. ingrahamii* enzyme showed decreased thermostability and high specific activity at low temperature, both of which are typical features of cold adapted enzymes. Furthermore, it was a more efficient biocatalyst compared to *E. coli* serine hydroxymethyltransferase (SHMT) particularly for side reactions. Many β-hydroxy-α-amino acids are SHMT substrates and represent important compounds in the synthesis of pharmaceuticals, agrochemicals and food additives. Thanks to these attractive properties, this enzyme could have a significant potential for biotechnological applications.

## 1. Introduction

Life, particularly microbial life, has evolved the capacity to proliferate in a broad range of different thermal environments. Thermal adaptation, particularly to extremes, has also limited the range of temperatures any individual organism may tolerate. This is illustrated for hot environments by a member of the Archaea dubbed strain 121, which was isolated from a deep sea hydrothermal vent and is capable of and restricted to growth at temperatures between 85 °C and 121 °C [1]. At the opposite thermal extreme, psychrophilic (cold adapted) microorganisms have been described that are capable of metabolizing in snow and ice at −20 °C, and numerous psychrophilic isolates have been characterized by their ability to proliferate at ≤0 °C and are restricted to *<*30 °C [2–5]. Clearly, mechanisms have evolved that provide the ability of an organism to adapt to a particular thermal environment. All these organisms are at thermal equilibrium with their environment, and all components of their cells must be suitably adapted to the cold [5,6]. Their phylogenetic diversity underscores the potential mechanistic variety that may have evolved to enable cold adaptation, and to some degree, cell-specific adaptation strategies have been identified. For example, psychrophilic organisms need enzymes able to efficiently catalyze viable reactions at temperatures close to 0 °C, at which most other species cannot grow because of their inability to maintain adequate metabolic fluxes [3,7,8]. Compared to their mesophilic and thermophilic counterparts, these enzymes display improved catalytic efficiency at low temperatures that is reflected in their higher turnover number (rise in *k*_cat_) and/or in the increased affinity for the substrate (reduction of *K*_M_). It is generally assumed that the structural determinants of such kinetic features are responsible not only for the low temperature optima but also for the inactivation of cold adapted enzymes at moderate temperatures (usually >40 °C). It has been proposed that thermolability of psychrophilic enzymes is a consequence of the higher flexibility of their molecular structure, in contrast with the thermostability of enzymes from thermophiles that, conversely, is correlated with rigidity of their polypeptide chain [3]. Thanks to recent advances provided by X-ray crystallography, structure modeling, protein engineering and biophysical studies, the adaptation strategies are beginning to be understood although structural and functional information still remain scant [9].

This study reports on the purification of recombinant serine hydroxymethyltransferase (SHMT; EC 2.1.2.1) from *Psychromonas ingrahamii* (*pi*SHMT), a bacterium recently isolated from the Arctic polar sea ice [10] able to grow in the temperature interval from −12 °C to 10 °C, and on the characterization of its structural and functional properties. SHMT is a pyridoxal-5′-phosphate (PLP)-dependent enzyme belonging to the fold type I superfamily which catalyzes the reversible conversion of l-serine and tetrahydropteroylglutamate (H_4_PteGlu) to glycine and 5,10-methylenetetrahydropteroylglutamate (5,10-CH_2_-H_4_PteGlu), respectively. It plays a central role in the one-carbon unit metabolism and it is a potential target for cancer therapy [11]. SHMT also catalyzes the H_4_PteGlu-independent cleavage of many 3-hydroxyamino acids and the decarboxylation of aminomalonate, at rates similar to that of H_4_PteGlu-dependent serine cleavage. This enzyme is structurally conserved after divergent evolution and it is ubiquitous in nature; many sequences from Eucarya, Eubacteria and Archaea are available in databanks as well as several unique crystal structures. In previous works, the molecular basis of the SHMT adaptation to low [12] and high [13,14] temperatures, using experimental and comparative *in silico* approaches, it has been analyzed. On the basis of this analysis, we decided to characterize the SHMT from *Psychromonas ingrahamii* since this bacterium displayed to lowest growth temperature among the ten available psychrophilic species [12]. To the best of our knowledge, only another PLP enzyme of fold type-I from a psychrophilic organism has been characterized, namely aspartate aminotransferase from *Pseudoalteromonas haloplanktis* [15]. In addition to this, further motivation to undertake the study of this enzyme resides in the fact that many β-hydroxy-α-amino acids that are substrates for SHMT, are important compounds for the synthesis of pharmaceuticals, agrochemicals and food additives [16]. Under these perspectives, the characterization of the kinetic and stability properties of SHMT from *P. ingrahamii* was considered attractive and interesting. To highlight the characteristics coupled to the adaptation to low temperature, the properties of the psychrophilic enzyme have been contrasted with those of the homologous counterpart from *E. coli*, which has been structurally and functionally characterized in depth and with which it shares 75% amino acid sequence identity. The structure of SHMT is, in fact, typically very conserved during evolution [12].

## 2. Results and Discussion

### 2.1. Heterologous Expression and Purification of the Recombinant *Psychromonas ingrahamii* SHMT

The gene coding for *Psychromonas ingrahamii* SHMT (*pi*SHMT) was synthesized from GENEART after code optimization. The codon usage was adapted to the codon bias of *E. coli* genes. The optimized gene should therefore allow high and stable expression rates in *E. coli*. We introduced this synthetic gene in the cloning/expression region of the vector pET28a (Novagen Inc.) in frame with His-Tag sequence at the *N*-terminal of the protein. The His-Tag sequence, which binds to divalent cations (Ni^2+^) immobilized on the His-Bind^®^ metal chelating resin, provides a convenient means of protein purification. Using this chromatographic procedure, we obtained approximately 100 mg of pure protein starting from 2 L of bacterial culture. The final protein concentration was about 0.1 mM. All the protein is purified with PLP bound, as judged by the comparison of the PLP concentration, determined from the absorbance at 388 nm after the PLP release from SHMT in 0.2 M NaOH, to the concentration of SHMT determined by absorbance at 280 nm.

### 2.2. Spectroscopic Studies

**Figure 1:**
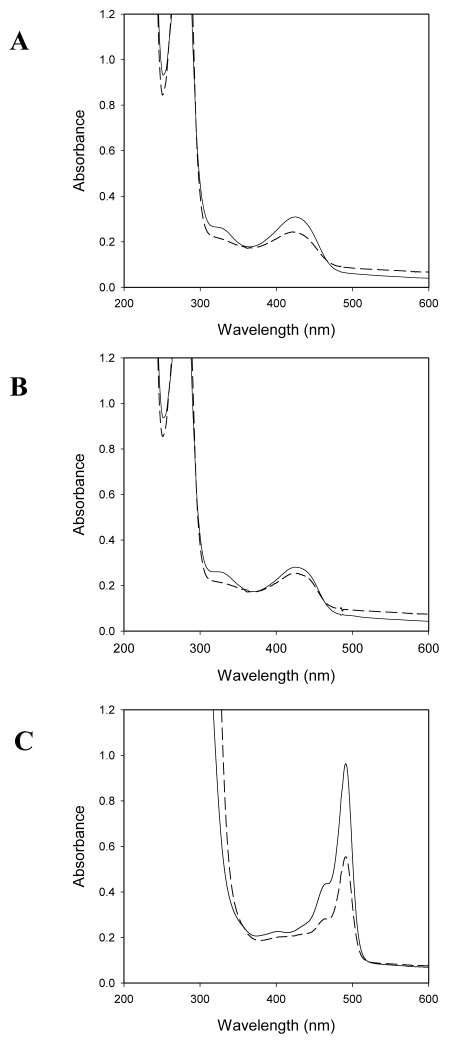
Absorbance spectra of *Psychromonas ingrahamii* SHMT (*pi*SHMT) and *Escherichia coli* SHMT (*e*SHMT) in the absence and presence of substrates. (**A**) Panel A shows the absorption spectra of 30 μM *pi*SHMT (continuous line), and *e*SHMT (dashed line) at 30 °C; (**B**) Panel B shows the absorption spectra after the addition of 90% saturating glycine (continuous line *pi*SHMT and dashed line *e*SHMT); (**C**) In panel C are shown the spectra taken after the addition of 100 μM H_4_PteGlu to the samples containing glycine.

The *e*SHMT exhibits a single absorption band in the visible region of the spectrum, with maximum intensity at 422 nm, typical of the protonated internal aldimine of PLP [17]. The *pi*SHMT absorbance spectrum is very similar to the *e*SHMT spectrum. Both exhibit a major band at 422 nm and a minor band at 334 nm. The addition of 90% saturating glycine to both SHMTs determined the increase of the 422 nm absorbing band, corresponding to the formation of the external aldimine, and the appearance of a band with a maximum absorbance at 343 nm, interpreted as the *gem-*diamine intermediates [17]. When H_4_PteGlu was added to the enzyme solution containing glycine, another band with maximum absorbance at 492 nm appeared, to the detriment of the external aldimine. It is known from literature that the 492 nm absorbing band corresponds to the accumulation of the quinonoid intermediate resulting from deprotonation of the Cα of glycine in the SHMT–Gly–H_4_PteGlu ternary complex [17]. The effect of H_4_PteGlu addition on the *gem-*diamine intermediate concentration is obscured by the intense absorption of this compound in the UV region. The two proteins, as shown in Figure 1, exhibit essentially the same spectral properties.

**Figure 2:**
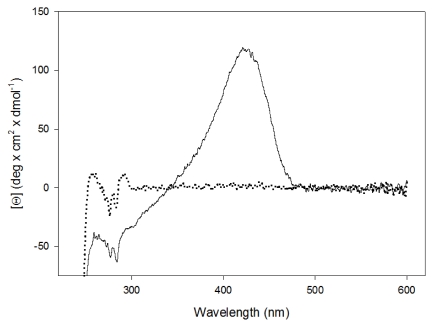
CD spectra of holo-*pi*SHMT (continuous line) and apo-*pi*SHMT (dotted line). CD spectra were recorded on 30 μM enzyme samples at 20 °C, in 20 mM potassium phosphate pH 7.2, containing 0.2 mM DTT, 0.1 mM EDTA and 50 mM Na_2_SO_4_.

The far UV CD spectra of *P. ingrahmii* enzyme at 2.5 μM subunit concentration is virtually identical to that of the *E. coli* enzyme, indicating that the two proteins have essentially the same secondary structure content (data not shown). The visible CD spectrum of *pi*SHMT is characterized by a positive cofactor band centered at around 422 nm. In the near UV region, were the ellipticity becomes negative, a broad band between 320 and 340 nm, also attributable to the cofactor, is visible. Below 310 nm, the spectrum shows the characteristic CD bands of aromatic residues [18]. Concerning the apo-form of the enzyme, the near UV CD spectrum is profoundly different with respect to that of the holo-form (Figure 2). These results could be due to the possibility that the loss of the cofactor induces some consistent change in the tertiary structure of the protein.

### 2.3. Thermal Stability

**Figure 3:**
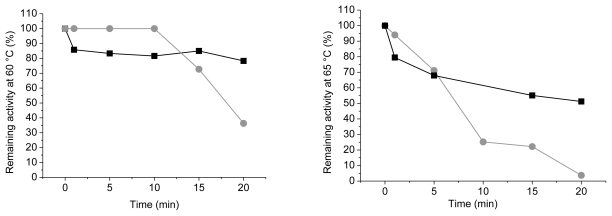
Residual enzyme activity after incubation at 60 °C (left panel) or 65 °C (right panel). Black and gray symbols refer to *Escherichia coli* and *Psychromonas ingrahamii* SHMT respectively. The reaction tested was: l-*allo*threonine cleavage assay. The values are the average of three independent replicates on different bathes of enzyme which did not vary more than 5%.

Both enzymes were incubated separately at 60 °C and 65 °C for different time intervals at the end of which the residual enzyme activity was measured. Figure 3 shows that *e*SHMT activity did not change significantly after the incubation time range employed (20 min) at 60 °C. On the contrary, the *P. ingrahmii* enzyme lost 30% and 70% of activity after 15 and 20 min, respectively, of incubation time at 60 °C. When the enzymes were incubated at 65 °C, a decrease of the *e*SHMT activity at about 50% of the starting activity after 15 and 20 min time intervals was observed. In the same conditions, *pi*SHMT activity decreased to about 20% and 3% of the initial activity, respectively. These results suggest that the activity of the mesophilic enzyme is resistant to incubation at moderately high temperature whereas the psychrophilic enzyme is quickly and irreversibly inactivated.

### 2.4. Catalytic Properties of *pi*SHMT

SHMT utilizes a wide variety of 3-hydroxyamino acid substrates and catalyzes numerous side reactions including decarboxylation, transamination and racemization. Table 1 reports the kinetic parameters of four reactions catalyzed by *pi*SHMT and *e*SHMT, at 30 °C. Except for the serine hydroxymethyltransferase reaction, for which the two enzymes exhibit the same kinetic parameters, the *k*_cat_ for l-*allo*threonine and l-*treo*phenylserine cleavage reactions catalyzed by *pi*SHMT, show a 3- and 5-fold increase, respectively. On the other hand, the *K*_M_ values for the same reactions do not change significantly. Catalytic efficiencies with respect to l-*allo*threonine and l-*treo*phenylserine substrates are increased at the same extent.

### 2.5. Thermal Denaturation Experiments

**Figure 4:**
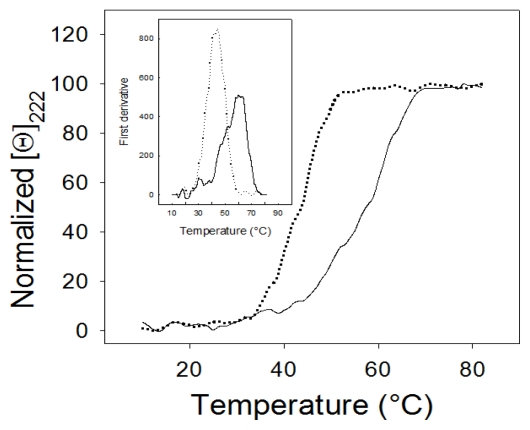
Thermal denaturation of apo- (dotted curve) and holo- (continuous curve) *Psychromonas ingrahamii* SHMT. The inset shows the first derivative of the same data.

Thermal stability of *pi*SHMT was investigated by monitoring the change of ellipticity at 222 nm in the far-UV CD spectrum, when the temperature was increased from 10 °C to 80 °C. The observed sigmoid transition was irreversible and the CD spectrum measured at the end of the cooling phase differed from that of the protein at 20 °C. The parameter chosen to compare the transition curves was the apparent melting temperature (Tm), defined as the mid-point of the sigmoid denaturation process and calculated by plotting the first derivative of the molar ellipticity values as a function of temperature. The experiments were performed on either holo- or apo-form of the enzyme (Figure 4). The apparent Tm value for the holo- and the apo-*p*iSHMT was 62.0 °C and 42.0 °C, respectively whereas the difference of the apparent Tm between the holo- and apo-*e*SHMT was 10.7 °C [21].

### 2.6. Temperature Dependence of Enzyme Activity

**Figure 5:**
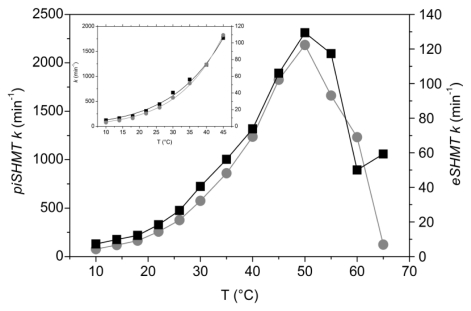
Temperature dependence of enzyme activity of *pi*- (gray symbols and left axis scale) and *e*- (black squares and right axis scale) SHMTs. Activities were measured for the retroaldol cleavage of l-*threo*-phenylserine. The values are the average of three independent replicates on different batches of enzyme which did not vary more than 2%. Inset: fitting of activity values of *pi-* and *e-*SHMTs to the Arrhenius equation in the temperature range 10–45 °C. Values refer to the same respective scales.

The temperature dependence of enzyme activity in the retroaldol cleavage of l-*threo*-phenylserine shows that *pi*SHMT and *e*SHMT have an optimal temperature at 50 °C (Figure 5). Calculated activation energies for *pi*SHMT and *e*SHMT are 15.4 ± 0.14 kcal mol^−1^ and 13.4 ± 0.13 kcal mol^−1^, respectively, confirming the similar mechanistic behaviour of both enzymes (Figure 5 inset). Our results show that, in the temperature range tested, the *pi*SHMT activity is at least ten fold higher than *e*SHMT activity.

### 2.7. Psychrophilicity of *Psychromonas ingrahamii* SHMT

Low thermal stability and high specific activity at low temperature are generally reported as the main features of cold adapted extracellular enzymes [22,23]. Our results suggest that *pi*SHMT is indeed more unstable than *e*SHMT, according to the rapid loss of activity after incubation at 60 °C or 65 °C (Figure 3) and to its apparent melting temperature, lower than that of the *e*SHMT [21]. Interestingly, the apoenzyme is far more unstable than the holoenzyme (Figure 4). This observation suggests that the intrinsic instability of the active site is partly compensated by the interaction with the cofactor. Flexibility of the active site is strongly supported by the near UV CD changes observed upon PLP binding (Figure 2). The instability and the consequent flexibility of the active site may be also functionally relevant for the conformational transitions during the low temperature transfer of the PLP to its binding site within the apoenzyme [24,25].

Our results clearly suggest that the *P. ingrahamii* SHMT is in general a more efficient biocatalyst compared to *E. coli* SHMT, in particular for the side reactions involving several substrates such as β-hydroxy-α-amino acids (Table 1), which represent important compounds in pharmaceuticals, agrochemicals and food additives [26].

The relative high activity characterizing psychrophilic enzymes is the main adaptive parameter to low temperatures and seems to be achieved by the destabilization of the active site or of the entire protein structure, allowing the catalytic center to be more flexible at low temperatures. In this way, it should be able to reach the transition complex with lower requirement of energy, generally not abundant in a low temperature environment. However, the relationships between activity, flexibility and stability still remain controversial. Overall, it seems that each psychrophilic enzyme adopts its own adaptive strategy [27–30]. We should mention that in a recent *in silico* analysis we have carried out on the structural adaptation of psychrophilic SHMTs, it turned out that a significant increase of frequency of flexible residues was consistently observed [12]. The *pi*SHMT activity is heat labile, as demonstrated by heat inactivation experiments. However, the curve representing the thermal dependence of the specific activities shows that the apparent maximal activity of *pi*SHMT is approximately the same as the mesophilic enzyme (Figure 5). In general, mesophilic and thermophilic enzymes show a temperature of maximal activity corresponding to the unfolding transition, suggesting that the observed loss of activity is due to protein unfolding. Interestingly, this is not the case for SHMT: mesophilic enzyme shows an apparent maximal activity (50 °C) below that at which the protein unfold, as the psychrophilic form. This result could be due to the particular catalytic apparatus evolved by SHMT, which is also responsible of its typical catalytic promiscuity. Furthermore, *pi*SHMT is 10 to 20 fold more active than *e*SHMT over the range of tested temperatures. Interestingly, activity of *pi*SHMT at 10 °C for the tested reaction is of the same order of magnitude of the activity of *e*SHMT at 37 °C.

## 3. Experimental Section

### 3.1. Materials

Ingredients for bacterial growth were from Difco. Chemicals for the purification of the enzymes were from BDH, Ni-NTA agarose was purchased from Sigma-Aldrich. All buffers, amino acids, and coenzymes used in the purification and assay of enzymes were of the highest purity available. Enzymes used in cloning procedures were purchased from New England BioLabs Inc. SHMT from *E. coli* was expressed and purified as previously described [20]. (6*S*)-H_4_PteGlu was a gift from A. G. Eprova, Schaffhausen, Switzerland. All other reagents were from Sigma-Aldrich.

### 3.2. Cloning of the Gene Encoding the *Psychromonas ingrahamii* SHMT

The coding sequence of the *pi*SHMT gene was synthesized by GENEART (Geneart AG, Regensburg, Germany), which provided us the 1290 bp DNA fragment, coding the protein, cloned in the pMK-RQ vector. The gene sequence properties were optimized for expression in the adopted system. We inserted the synthetic gene into the pET28a(+) expression vector (Novagen, Inc., Madison, WI, USA) between the restriction sites *Nde*I and *Eco*RI. The pET28-*pi*SHMT construct was used to transform *E. coli* HMS174(DE3) strain cells (Novagen, Inc.). The pET28a(+) vector adds 20 residues to the *N*-terminal of *pi*SHMT, including a sequence of six His residues for binding to an Ni-NTA column. The sequence of the DNA insert was verified in both directions.

### 3.3. Expression and Purification of *Psychromonas ingrahamii* SHMT

An overnight culture (40 mL) of HMS174 (DE3) cells, transformed with the *pi*SHMT overexpressing plasmid, was inoculated into 2 L of Luria-Bertani broth containing kanamycin (40 μg/mL). Bacteria were grown aerobically at 37 °C to exponential phase (until the optical density at 600 nm was 0.3–0.4), then the incubation temperature was lowered to 25 °C and the expression of *pi*SHMT was induced with 0.05 mM isopropyl thio-β-d-galactoside (IPTG). Bacteria were harvested after 20 h and suspended in 10 mM Tris/HCl buffer, pH 7.6, containing 1 mM EDTA. Cell lysis was carried out by the addition of 10 mg of lysozyme per gram of packed bacterial cells and incubation for 20 min at room temperature. A 25 μL aliquot of protease inhibitor cocktail for bacterial extracts (Sigma) was added and the cell extract was frozen. After defreezing and the addition of 1% (w/v) streptomycin sulfate to precipitate DNA, the cell extract was centrifuged at 15,000 g for 30 min and the pellet was discarded. Solid ammonium sulfate was added to the cell extract to 35% of saturation. The solution was centrifuged at 15,000 g for 20 min and the pellet was discarded. Ammonium sulfate was added to the supernatant to 75% of saturation and the precipitated protein was collected by centrifugation as before. The protein pellet was dissolved in a minimal amount of 20 mM potassium phosphate, pH 7.2, and dialyzed for 4 h against 1 L of the same buffer, with a buffer change against 2 L of the same buffer overnight. After a brief centrifugation to remove precipitate materials, the dialysed material was added to an Ni-NTA column (3 cm × 8 cm) equilibrated with 20 mM sodium phosphate, pH 7.4 containing 300 mM NaCl. The enzyme binds to the column as a yellow band. The column was washed with equilibration buffer until the absorbance at 260 nm was below 0.1 and then with the same buffer with increasing concentration of imidazole (5–10–25 mM). The enzyme was eluted with 500 mM imidazole. Fractions were collected, and those absorbing at 422 nm were pooled and concentrated by 75% ammonium sulfate precipitation, followed by dialysis in 20 mM potassium phosphate at pH 7.0, containing 200 μM DTT and 100 μM EDTA. All procedures were performed at about 4 °C. The purity of the *pi*SHMT sample was checked by SDS-PAGE and judged to be ≥98%. The protein concentration was determined measuring the absorbance at 280 nm and using a ɛ_280_ = 43.320 M^−1^ cm^−1^ calculated according to Gill and von Hippel [31].

### 3.4. Preparation of Apoenzyme

Apo-*pi*SHMT was prepared using l-cysteine as previously described [24]. A small, residual fraction (less than 5%) of holoenzyme, estimated by activity assays, was present in the apoenzyme samples. The same observation was also made with the mesophilic enzyme. This residual activity could be due to incomplete removal of PLP. The holoenzyme was reconstituted by incubating the apoenzyme with PLP and separating the unbound PLP from the enzyme by dialysis.

### 3.5. Spectroscopic Measurements

UV-visible CD spectra were recorded with a Jasco 725 spectropolarimeter in the circular dichroism mode. Far (190–250 nm), near UV (250–310 nm), and visible (310–500 nm) CD spectra were measured using 0.2 and 1.0 cm path length quartz cuvettes. UV-visible spectra and kinetic measurements in the activity assays were recorded on a Hewlett-Packard 8453 diode-array spectrophotometer equipped with a thermostatically controlled water bath. All spectroscopic measurements were carried out at 20 °C in 20 mM potassium phosphate pH 7.2, containing 0.2 mM DTT and 0.1 mM EDTA.

### 3.6. Activity Assays

All assays were carried out in 20 mM potassium phosphate pH 7.2, containing 0.2 mM DTT and 0.1 mM EDTA at 30 °C. The serine hydroxymethyltransferase activity was measured with 0.05 μM enzyme samples with l-serine and H_4_PteGlu as substrates, using a coupled assay as previously described [20]. In order to determine the *K*_M_ for l-serine, H_4_PteGlu was maintained at 0.03 mM and the l-serine concentration was varied between 0.06 mM and 25 mM.

The rate of l-*allo*-threonine cleavage (3 μM enzyme samples) was followed by determining the rate of reduction of the product acetaldehyde by alcohol dehydrogenase and NADH at 340 nm [32].

Benzaldehyde production from phenylserine cleavage was measured spectrophotometrically at 279 nm, employing a molar absorption value of ɛ_279_ = 1400 cm^−1^·M^−1^ [33].

### 3.7. Data Analysis

All data analyses were carried out using the software Prism [34] and OriginPro8 [35].

### 3.8. Thermal Denaturation Experiments

The protein samples (2.3 μM) in 50 mM sodium Hepes buffer, pH 7.2, containing 0.2 mM DTT and 0.1 mM EDTA were heated from 10 °C to 80 °C with a heating rate of 1 degree min^−1^ controlled by a Jasco programmable Peltier element. The dichroic activity at 222 nm was monitored continuously every 0.5 °C. All thermal scans were corrected for solvent contribution at the different temperatures. Melting temperature values were calculated by taking the first derivative of the ellipticity at 222 nm with respect to temperature [36]. Replicate determinations of melting temperature values with different batches of enzyme did not vary more than 2%.

### 3.9. Heat Inactivation

*e*SHMT or *pi*SHMT was kept in a thermostatically controlled block at 60 °C and 65 °C. At different time intervals (0–20 min) aliquots of 30 mL were withdrawn and chilled in ice. These aliquots were subsequently assayed at 30 °C for residual enzyme activity, employing the l-*allo*-threonine cleavage assay, after adding the remaining components of enzyme assay mixture. Results of heat inactivation experiments were expressed as percent activity remaining compared to the control value obtained at zero time of incubation.

### 3.10. Temperature Dependence of Enzyme Activity

The temperature dependence of the rate of retroaldol cleavage of l-*threo*-phenylserine was determined, using either *e*SHMT or *pi*SHMT as catalyst, over the range from 10 °C to 65 °C. The steady state velocity of the reaction was measured using 15 mM substrate. Data in range temperature 10–45°C were used in a global fit to the Arrhenius equation in which the activation energy was a shared parameter.

## 4. Conclusions

In conclusion, our studies demonstrate that SHMT from *Psychromonas ingrahamii* is an enzyme with psychrophilic features, which deserves further investigations. This enzyme has a lower melting temperature compared to its counterpart, although it is not as unstable as other psychrophilic enzymes reported in the literature, despite being substantially more active than the *e*SHMT. Therefore this enzyme appears, in a sense, optimized for good stability combined with good activity at low temperature. This suggests also that *P. ingrahamii* may be a source of enzymes with attractive characteristics for biotechnological applications.

## Figures and Tables

**Table 1:** Comparison of the kinetic parameters of several reactions catalyzed by *Psychromonas ingrahamii* SHMT and *Escherichia coli* SHMTs at 30 °C.

Reactions	*Psychromonas ingrahamii*			*Escherichia coli*		

Substrate	*K*_M_ (mM)	*k*_cat_ (min^−1^)	*k*_cat_/*K*_M_ (min^−1^ mM^−1^)	*K*_M_ (mM)	*k*_cat_ (min^−1^)	*k*_cat_/*K*_M_ (min^−1^ mM^−1^)
l-threonine	20.2	6.6	0.3	43 a	4.3 a	0.1 a
l-*threo-*phenylserine	17.2	852	50	19 a	167 a	8.8 a
l-*allo-*threonine	1.6	107	67	1.5 b	30 b	20 b
l-serine (H_4_PteGlu 30 μM)	0.4	555	1388	0.3 b	640 b	2130 b

a from reference [19];

b from reference [20].
